# Diabetes is associated with a poor prognosis in patients with psoriasis and coronary artery disease

**DOI:** 10.1186/s12902-025-01996-z

**Published:** 2025-07-14

**Authors:** Lin Zhao, Lin Sun, KunQi Yang, ZengLei Zhang, ZuoZhi Li, Man Wang, XianLiang Zhou, Yan Zeng, WeiXian Yang

**Affiliations:** 1 Department of Integrative Medicine Cardiology, China-Japan Friendship Hospital, No. 2 Yinghua East Street, Beijing, China; 2 Department of Cardiology, Fuwai Hospital, National Center for Cardiovascular Disease, Chinese Academy of Medical Sciences and Peking Union Medical College, No.167, Beilishi Road, Beijing, China

**Keywords:** Diabetes, Major adverse cardiovascular events, Coronary artery disease, Psoriasis, Prognosis

## Abstract

**Background:**

There is an increased risk of diabetes and cardiovascular disease among patients with psoriasis. However, whether diabetes affects the cardiovascular adverse events in patients with psoriasis who have suffered from coronary artery disease remains unclear. This study aimed to explore the prognostic role of diabetes in this particular population.

**Methods:**

This single-center, retrospective cohort study included all consecutive adult patients with psoriasis and coronary artery disease admitted at our hospital between January 2017 and May 2022. Clinical records were collected and compared between patients with and without diabetes. Survival curves were derived using Kaplan–Meier methods. Multivariable Cox regression was used to control potential confounding.

**Results:**

This study included 305 participants, including 147 patients (48.2%) with diabetes. Patients with diabetes were more likely to have hypertension (*p* = 0.045), peripheral vascular disease (*p* = 0.043) and the history of stroke (*p* = 0.041). Patients with diabetes also had higher levels of low-density lipoprotein cholesterol (*p* = 0.039) and homocysteine (*p* = 0.006). After a median follow-up of 36 months, patients with diabetes had a higher incidence of major adverse cardiovascular events (MACE) than patients without diabetes (*p* = 0.032). According to the results of the Cox regression analysis, only diabetes (*p* = 0.039) was associated with MACE. The subgroup analysis showed that diabetes was associated with MACE, especially in male patients (*p* = 0.008) and those without chronic kidney disease (*p* = 0.021).

**Conclusion:**

In patients with psoriasis and coronary artery disease, diabetes is independently linked with MACE. These findings will help the risk assessment for patients with psoriasis and coronary artery disease.

**Supplementary Information:**

The online version contains supplementary material available at 10.1186/s12902-025-01996-z.

## Background

Psoriasis is a chronic inflammatory disease mediated by the immune system, which is characterized by keratinocyte hyperproliferation and T-lymphocyte inflammation [1]. It affects 2–3% of the global population [1, 2]. There were approximately 4.62 million incident cases of psoriasis worldwide in 2019, with an age-standardized incidence rate of 57.8 per 100,000 people [2]. Various factors, including age, sex, geography, ethnicity, and environmental factors, contribute to the variation in disease prevalence [3, 4]. More than half of patients with psoriasis are estimated to present in the first three decades of their lives [5]. Psoriasis has traditionally been considered a skin disease, but it is now regarded as a systemic inflammatory disease accompanied by a wide range of co-morbidities [6]. Many metabolic disorders have increased incidences in patients with psoriasis, including diabetes [5, 7–9]. These diseases share similar underlying pathophysiology, such as genes changes, epigenetic changes, inflammation, environmental conditions, and insulin resistance [10].

So far, the factors affecting the prognosis of patients with psoriasis who have already suffered from coronary artery disease are still unclear. It has been reported that geographical differences exist in the clinical characteristics and management of these patients. In Chinese patients with psoriasis, comorbid cardiovascular disease is associated with smoking, obesity, hypertension, diabetes, and the severity of psoriasis [11]. Despite the substantial disease burden, the use of biologics in China remains limited due to financial burden [12]. In contrast, Western countries increasingly adopt an integrated care model involving dermatologists and cardiologists, with greater emphasis on cardiovascular screening and early intervention [13]. To date, it remains uncertain whether the presence of a major metabolic disorder, diabetes mellitus, influences the prognosis of patients with psoriasis and coronary artery disease. This study investigates the impact of diabetes on clinical outcomes in patients with psoriasis and coronary artery disease, based on real-world data.

## Methods

### Study population

A retrospective cohort analysis was conducted on all consecutive adult patients with psoriasis and coronary artery disease in Fuwai Hospital, Beijing, China, from January 2017 through May 2022. Clinical medical records of the patients at the first admission were included. Patients younger than 18 years of age, patients with a history of malignancies, or patients with a history of other rheumatic and connective tissue diseases were excluded from the study. Disease identification and exclusion were based on International Classification of Diseases, 10th Revision (ICD-10) codes recorded in the electronic medical records system. Specifically, psoriasis was identified using ICD-10 codes L40.0–L40.9, and coronary artery disease using codes I21–I25. Exclusion criteria included malignant neoplasms (ICD-10 codes C00–C97), rheumatic diseases (ICD-10 codes M05–M14), and connective tissue diseases (ICD-10 codes M30–M36). The Institute Ethics Committee of Fuwai hospital approved this study, which followed the Declaration of Helsinki. The data was anonymized before analysis.

### Biochemical analysis and definitions

Laboratory test results and imaging data during the patient’s first hospitalization were included. A minimum of 12 h of fasting was required for all patients before venous blood was collected. The biochemical measurements were performed at Fuwai Hospital’s clinical chemistry department. An automatic biochemistry analyzer (Hitachi 7150, Tokyo, Japan) was used to measure the levels of serum triglycerides, high-density lipoprotein cholesterol, low-density lipoprotein cholesterol (LDL-c) and total cholesterol. When multiple imaging studies or laboratory results were available, the earliest complete examination during the admission was selected. Information regarding culprit vessels and other angiographic characteristics was retrospectively extracted from the electronic medical records and coronary angiography reports. The data were reviewed and verified by two experienced interventional cardiologists independently, and discrepancies were resolved through discussion or adjudicated by a third reviewer. Culprit lesions were defined according to the interventional report and corresponding clinical presentation. These assessments were conducted retrospectively at the time of this study.

The diagnosis of diabetes was established either based on ICD-10 codes E10–E14 or laboratory test results in accordance with established clinical guidelines. Specifically, the diagnostic criteria included [14, 15]: (1) the presence of typical symptoms of diabetes combined with random blood glucose levels ≥ 11.1 mmol/l; or (2) in asymptomatic patients, a fasting plasma glucose level of ≥ 7.0 mmol/l, or a 2-hour plasma glucose level of ≥ 11.1 mmol/l during 75 g oral glucose tolerance tests, or an glycosylated hemoglobin level of ≥ 6.5% confirmed by repeated tests; or (3) a previous diagnosis of diabetes and current treatment with hypoglycemic medication or diet. Hypertension was defined following this guideline [16]: (1) patients with systolic blood pressure of ≥ 140 mmHg and/or diastolic blood pressure of ≥ 90 mmHg in the office following repeated examinations; or (2) patients with a home systolic blood pressure of ≥ 135 and/or diastolic blood pressure ≥ 85 mmHg; or (3) a previous diagnosis of hypertension with established antihypertensive medication or diet. A major adverse cardiac event (MACE) was defined as a combination of all-cause death, unplanned revascularization, non-fatal stroke, re-hospitalization due to heart failure or severe arrhythmias and non-fatal myocardial infarction. In this study, the unplanned revascularizations were defined as repeat percutaneous coronary interventions or coronary artery bypass grafting performed for ischemic symptoms or objective evidence of ischemia in any segment of the target vessel [17]. Non-fatal myocardial infarction refers to ST-segment elevation myocardial infarction and non-ST-segment elevation myocardial infarction not resulting in death, diagnosed based on clinical symptoms, elevated cardiac biomarkers, and electrocardiography or imaging evidence. Unstable angina events were considered as MACE only if they led to urgent hospitalization. Non-fatal stroke events were confirmed by medical records and required radiological evidence. Re-hospitalization for heart failure was defined as a hospitalization in which heart failure was the primary diagnosis, based on clinical symptoms, elevated natriuretic peptides, and imaging evidence of cardiac dysfunction. Severe arrhythmias included sustained ventricular tachycardia, ventricular fibrillation, cardiac arrest requiring resuscitation, or new-onset atrial fibrillation/flutter requiring hospitalization or emergent management. All-cause death included both cardiac and non-cardiac deaths, ascertained from hospital records and/or official death certificates.

The Chronic Kidney Disease Epidemiology Collaboration equation was used to calculate the estimated glomerular filtration rate using [18], and chronic kidney disease (CKD) was considered when the patients had an estimated glomerular filtration rate < 60 ml/min/1.73m^2^ for at least three months [19]. All patients included in this analysis were completed at least 6 months of follow-up after discharge. Follow-up data were collected using standardized telephone interviews and review of outpatient medical records.

### Statistical analysis

Continuous values were reported as means ± standard or medians (25th, 75th percentiles) and categorical values were presented as numbers (percentages). Students’ t-tests or rank-sum tests were used to compare continuous values between groups, and differences of categorical values were detected using Pearson’s chi-square or Fisher’s exact tests. We constructed Kaplan-Meier survival curves and used log-rank tests to compare the groups. Subgroup analyses of MACE were performed based on the following factors: age (< 60 and ≥ 60 years), gender, hypertension, and CKD. In each subgroup analysis, the link between diabetes and MACE was evaluated using Cox regression analysis. The multivariable Cox regression analysis included parameters with *p* < 0.1 in the univariate Cox regression analysis. For survival analysis, the start date for survival analysis was defined as the discharge date of the index hospitalization. The event date was defined as the date on which the first MACE occurred during the follow-up period. Patients who did not experience any MACE were censored at the last available date of follow-up, either from outpatient visits or telephone interviews. As this study was based on a prespecified hypothesis regarding the association between diabetes and MACE, no adjustment for multiple comparisons was applied. A two-tailed p-value of < 0.05 was considered statistically significant. SPSS 25.0 (IBM Corp., Armonk, NY, USA) was used for all statistical analyses. The Kaplan–Meier curve and forest plot were generated using GraphPad Prism 8.0 (GraphPad, San Diego, CA, USA). Since no previous studies have reported the effects of diabetes on the prognosis of patients with psoriasis and coronary artery disease, we included all patients who met the inclusion criteria in our hospital between January 2017 and May 2022. This study included 311 patients, of which 6 had incomplete clinical data. Consequently, 305 patients were included in the study for analysis, of which 158 were without diabetes and 147 were with diabetes. The incidence of MACEs was 17.2% in the non-diabetes group and 32.1% in the diabetes group. We set α to 0.05 and calculated a power of 86.3% by using PASS (Power Analysis and Sample Size, version 15.0).

## Results

### Clinical characteristics of the patients

Of the 305 participants, 147 (48.2%) met the criteria for diabetes. There were no significant differences in age (*p* = 0.073), sex (*p* = 0.801), body mass index (*p* = 0.061), and CKD (*p* = 0.403) between the two groups. More patients in the diabetes group had hypertension (62.6% vs. 51.9%, *p* = 0.045), a history of stroke (12.2% vs. 5.7%, *p* = 0.041), and peripheral vascular disease (7.5% vs. 2.5%, *p* = 0.043) than those in the non-diabetes group (Table 1). Patients with diabetes had higher levels of LDL-c [2.33 (1.82,2.95) mmol/l vs. 2.14 (1.65,2.78) mmol/l, *p* = 0.039] and homocysteine [15(12,21) vs. 13(11,17) µmol/l, *p* = 0.006] than those without diabetes. Patients in the diabetes group were more likely to have right coronary artery involvement (78.2% vs. 67.1%, *p* = 0.030). In contrast, there were no significant differences on the involvement of left anterior descending artery (*p* = 0.146), the involvement of left circumflex artery (*p* = 0.853) and the number of vessels involved between the diabetes and non-diabetes groups (Table 1).

**Table 1:** Baseline clinical characteristics of the patients

Parameter	Whole cohort (*n* = 305)	Non-Diabetes (*n* = 158)	Diabetes (*n* = 147)	*P*
Age, years (*n* = 305)	58.61 ± 9.81	57.68 ± 11.03	59.7 ± 8.64	0.073
Male, %(*n* = 305)	270(88.5)	140(88.6)	130(88.4)	0.801
Body mass index, kg/m^2^ (*n* = 305)	26.11 ± 3.30	25.82 ± 3.14	26.44 ± 3.54	0.061
Current smoker, % (*n* = 305)	203(66.6)	107(67.7)	96(65.3)	0.772
Hypertension, % (*n* = 305)	174(57.1)	82(51.9)	92(62.6)	0.045*
Hyperlipidemia, % (*n* = 305)	290(95.1)	148(93.7)	142(96.6)	0.117
Previous stroke, % (*n* = 305)	27(8.9)	9(5.7)	18(12.2)	0.041*
Peripheral vascular disease, %(*n* = 305)	15(4.9)	4(2.5)	11(7.5)	0.043*
CKD, %(*n* = 305)	27(8.9)	12(7.6)	15(10.2)	0.403
ACS, %(*n* = 305)	194(63.6)	101(63.9)	93(63.3)	0.98
Psoriasis characteristics				
Psoriatic arthritis, % (*n* = 266)	10(3.8)	7(5.2)	3(2.3)	0.397
Topical treatment, % (*n* = 266)	174(65.4)	89(65.4)	85(65.4)	0.743
Phototherapy, % (*n* = 266)	33(12.4)	16(11.8)	17(13.1)	0.677
Biologic treatment, % (*n* = 266)	28(10.5)	18(13.2)	10(7.7)	0.165
Laboratory values				
Total cholesterol, mmol/L (*n* = 301)	3.81(3.17, 4.42)	3.83(3.26,4.56)	3.80(3.14,4.36)	0.212
Low-density lipoprotein cholesterol, mmol/L (*n* = 301)	2.23(1.73, 2.93)	2.14(1.65,2.78)	2.33(1.82,2.95)	0.039*
High-density lipoprotein cholesterol, mmol/L (*n* = 301)	1.04(0.87, 1.27)	1.10(0.94,1.29)	1.03(0.88, 1.22)	0.058
Triglycerides, mmol/L(*n* = 301)	1.50(1.03, 2.05)	1.44(1.09,1.97)	1.5(1.06,2.18)	0.387
Homocysteine, µmol/L(*n* = 235)	14(11, 18)	13(11,17)	15(12,21)	0.006*
eGFR, ml/min/1.73m^2^ (*n* = 305)	87.53 ± 19.85	87.88 ± 20.78	87.36 ± 19.31	0.818
Culprit vessel (*n* = 305)				
LAD, %	265(86.9)	133(84.2)	132(89.8)	0.146
LCX, %	208(68.2)	107(67.7)	101(68.7)	0.853
RCA, %	221(72.5)	106(67.1)	115(78.2)	0.030*
No. of diseased vessels (*n* = 305)				
1, %	67(22.0)	40(25.3)	27(18.4)	0.143
2, %	75(24.6)	39(24.7)	36(24.5)	0.969
3, %	159(52.1)	76(48.1)	83(56.5)	0.144
PCI, % (*n* = 305)	177(58.0)	91(57.6)	86(58.5)	0.773
CABG, % (*n* = 305)	23(7.5)	11(7.0)	12(8.2)	0.668
MACE, %(*n* = 291)	71(24.4)	26(17.2)	45(32.1)	0.032*

Abbreviations: ACS, acute coronary syndrome; CABG, coronary artery bypass grafting; CKD, chronic kidney disease; eGFR: estimated glomerular filtration rate; LAD, left anterior descending artery; LCX, left circumflex artery; MACE, major adverse cardiovascular events; PCI, percutaneous coronary intervention; RCA, right circumflex artery. * *p* < 0.05

### Clinical outcomes of the patients

**Fig. 1:**
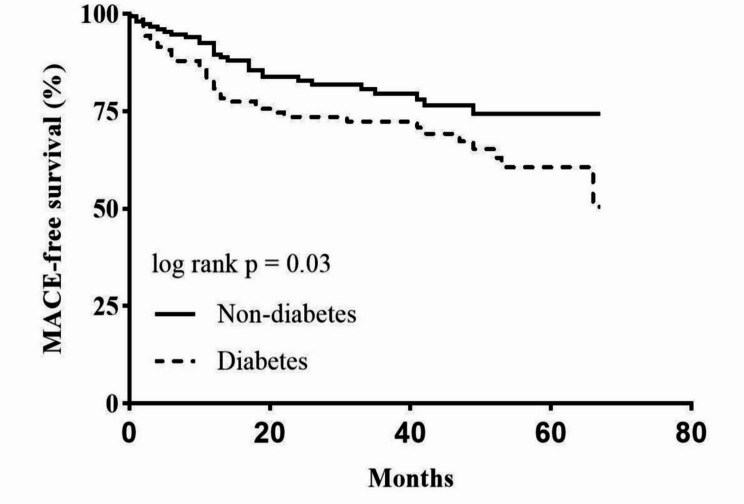
Kaplan-Meier survival curves estimated MACE for patients with and without diabetes in the whole cohort. MACE indicates major adverse cardiovascular events

A total of 291 patients (95.4%) were followed up for a median of 36 months (interquartile range [IQR]: 20–52), including 151 patients without diabetes and 140 patients with diabetes. The median follow-up time was 33 months (IQR: 19–52) in the diabetes group and 37 months (IQR: 21–52) in the non-diabetes group. The 14 patients (4.6%) lost to follow-up were excluded from the final analysis. Finally, 45 patients (32.1%) in the diabetes group and 26 patients (17.2%) in the non-diabetes group had MACE, and there was a significant difference between the diabetes and non-diabetes groups (*p* = 0.032). Kaplan–Meier survival curves also showed a significant difference in MACE (log-rank *p* = 0.030) between the diabetes group and non-diabetes group (Fig. 1). According to the results of the multivariable Cox regression analysis, diabetes was the only variable significantly associated with MACE (hazard ratio [HR] = 1.661, 95% confidence interval [CI]: 1.025–2.692; *p* = 0.039) (Table 2).

**Table 2:** The results of COX regression analysis

Parameter	Univariate cox regression analysis			Multivariate cox regression analysis		
	HR	95%CI	*p*	HR	95%CI	*p*
Age	0.985	0.962–1.008	0.196			
Male	0.577	0.316–1.054	0.074	0.633	0.339–1.182	0.152
Body mass index	1.053	0.978–1.133	0.170			
Diabetes	1.675	1.043–2.689	0.033*	1.661	1.025–2.692	0.039*
Current smoker	0.836	0.518–1.351	0.465			
Hypertension	1.052	0.657–1.683	0.834			
Hyperlipidemia	0.933	0.375–2.320	0.881			
Previous stroke	1.039	0.448–2.419	0.902			
Peripheral vascular disease	1.398	0.421–3.951	0.472			
CKD	1.492	0.714–3.117	0.287			
ACS	1.047	0.646–1.698	0.851			
Psoriatic arthritis	1.946	0.704–5.378	0.199			
eGFR	0.997	0.986–1.009	0.643			
Total cholesterol	0.966	0.766–1.217	0.769			
Low-density lipoprotein cholesterol	1.152	0.901–1.474	0.260			
High-density lipoprotein cholesterol	1.131	0.622–2.057	0.686			
Triglyceride	1.204	0.97–1.494	0.092	1.154	0.930–1.432	0.193
Homocysteine	0.994	0.972–1.026	0.584			
Culprit vessel						
LAD	1.965	0.793–4.902	0.142			
LCX	1.137	0.661–1.912	0.605			
RCA	1.330	0.702–2.103	0.507			
No. of diseased vessels						
1	0.788	0.438–1.415	0.425			
2	0.968	0.561–1.672	0.908			
3	1.280	0.798–2.052	0.305			

Abbreviations: ACS, acute coronary syndrome; CKD, chronic kidney disease; eGFR: estimated glomerular filtration rate; HR, hazard-ratio; LAD, left anterior descending artery; LCX, left circumflex artery; RCA, right circumflex artery; 95%CI, 95% confidence interval. * *p* < 0.05

**Fig. 2:**
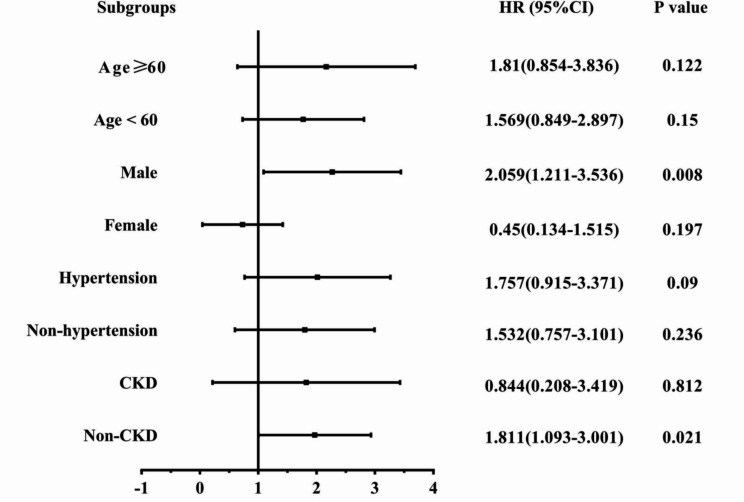
Subgroup analyses for the MACE HRS and 95% Cls were calculated by reference to the Non-diabetes group. MACE, major adverse cardiovascular events; CKD, chronic kidney disease; HR, hazard ratio; 95% CI, 95% confidence interval

In the subgroup analysis for MACE, significant differences were observed (Fig. 2). The association between diabetes and MACE was more pronounced among men (HR = 2.059, 95% CI: 1.211–3.536, *p* = 0.008) and patients without CKD (HR = 1.811, 95% CI: 1.093–3.001, *p* = 0.021). No significant differences were observed in patients with or without hypertension (*p* = 0.090 and 0.236, respectively), and those aged ≥ 60 years or < 60 years (*p* = 0.122 and 0.150, respectively) between the diabetes and non-diabetes groups. These results were consistent with that of Kaplan–Meier survival curves. According to the results of Kaplan-Meier survival curves, there was a significant difference in MACE among men (log-rank *p* = 0.006) between patients with and without diabetes; a difference in MACE (log-rank *p* = 0.019) was also observed between the diabetes and non-diabetes groups among patients without CKD (Supplementary Figure 1).

## Discussion

This study indicates that diabetes is associated with MACE in patients with psoriasis and coronary artery disease. Subgroup analysis show that diabetes is positively associated with MACE, especially in men and in those without CKD. Owing to the substantial economic burden and adverse effects associated with cardiovascular disease, high-risk patients require more stringent monitoring to achieve better prognoses.

The presence of psoriasis increases the risk of myocardial infarction, stroke, cardiovascular and all-cause mortality [20–22]. Cardiovascular disorders and psoriasis are likely related owing to shared inflammatory factors influenced by genetic and molecular pathways between the two diseases [23]. Shared chronic inflammatory factors have various effects on the endothelium, leading to proatherogenic phenotype production [23]. Unfortunately, studies on the risk factors associated with the cardiovascular adverse events in patients with both psoriasis and coronary artery disease are limited. This study explored the effects of diabetes on prognosis of these patients and discovered that diabetes is linked with cardiovascular adverse events in these patients. Given the observed association between diabetes and MACE in patients with psoriasis and coronary artery disease, our findings support the need for enhanced cardiovascular risk stratification in this population. Regular assessments of glycemic control, lipid levels, blood pressure, and renal function should be emphasized. In addition, the use of cardioprotective antidiabetic agents may be considered where appropriate. Early collaboration between dermatologists, cardiologists, and endocrinologists could help facilitate timely risk assessment and therapeutic optimization.

Patients with psoriasis have been reported to have an increased prevalence and incidence of diabetes [7–9]. Armstrong et al. described an overall increased prevalence of diabetes of 59% among patients with psoriasis and up to 97% among those with severe psoriasis [7]. Among studies that assessed incidence, diabetes was 27% more likely to develop in patients with psoriasis than those without [7]. Mamizadeh et al. [8] combined the results of 38 studies and confirmed that patients with psoriasis were with an increased risk of diabetes mellitus, in line with another meta-analysis [9]. Diabetes and psoriasis do not only share comorbidities but also share common pathophysiology. Genetic mechanisms are the most important pathophysiological links between these two diseases. Both psoriasis and diabetes are associated with the PTPN22, ST6GAL1, and JAZF1 genes [24]. Psoriasis and diabetes are also linked to the pleiotropic susceptibility locus CDKAL1 [25]. Moreover, insulin resistance is another important pathophysiological mechanism of both diseases [10]. Diabetes and psoriasis have more harmful effects on metabolism than either disease alone [26], and patients with both diseases are more prone to develop micro- and macrovascular complications [5]. Ramos et al. [27] revealed that abdominal aortic calcification was more prone to occur in patients with psoriasis than controls, and diabetes was an independent risk factor of abdominal aortic calcification in these patients. Moreover, abdominal aortic calcification could increase the risk of the cardiovascular disease and mortality [28, 29]. This could explain the role of diabetes on the occurrence of cardiovascular adverse events in patients with psoriasis and coronary artery disease in our study.

It is easy to understand that the proportions of patients with hypertension, stroke or peripheral vascular disease in diabetes group are higher than those in the patients without diabetes. Studies have reported that diabetes is an independent risk factor for cardiovascular disease, cerebrovascular disease, and peripheral arterial disease [30]. Hypertension is a common condition coexisting with diabetes and is positively associated with atherosclerotic cardiovascular disease [30].

In this study, patients with diabetes had higher levels of homocysteine and LDL-c than patients without diabetes, which is consistent with the results of a study by Brazzelli et al. [26]. In patients with diabetes mellitus, homocysteine level was also linked with all-cause and cardiovascular mortality [31]. Biologically, homocysteine contributes to cardiovascular disease by several mechanisms, including inducing damage to the vascular endothelium and arterial walls caused by oxidative stress and via its blood coagulant properties [32, 33]. LDL-c is also an important risk factor for cardiovascular disease because it plays a major role in atherosclerosis [34]. LDL-c is the primary lipid for predicting cardiovascular risk and is linked to increased mortality of cardiovascular diseases [35]. Patients with diabetes had higher homocysteine and LDL-c levels than those without diabetes, strengthening the contention that cardiovascular adverse events are more prone to occur in patients with diabetes.

In the subgroup analysis, MACE was associated with diabetes in men. One reason for the gender difference in subgroup analysis may be that compared with female patients with psoriasis, male patients with psoriasis have more adverse lifestyle habits, such as smoking and drinking [36]. In addition, male patients also have higher proportions of chronic diseases, including hypertension and hyperlipidemia [36], which could increase the risk of cardiovascular disease. Although psoriasis itself could increase the risk of CKD [37], care should be taken to screen for diabetes in patients with psoriasis and coronary artery disease who do not have CKD, because diabetes is linked with subsequent adverse events in these patients. It should be admitted that although we included all patients with psoriasis and coronary artery disease admitted at our hospital in the past 5 years, the number of female patients and patients with CKD was limited, which may influence the results of statistical calculation. Therefore, we need studies with larger sample sizes to accurately capture the prognostic effects of diabetes in these patients with different clinical characteristics.

Psoriasis is frequently accompanied by comorbidities such as type 2 diabetes, as described in the concept of the “psoriatic comorbidome” [38], which can impact disease severity and influence treatment choices. The presence of comorbid conditions may restrict the use of conventional systemic therapies like methotrexate due to concerns over hepatotoxicity, drug interactions, or immunosuppression [39], often leading to earlier use of biologics. For polymedicated, metabolically complex diabetic patients, personalized and holistic treatment strategies are essential. Recent studies support incorporating chronomedicine [40] and dietary considerations [41]. Treatment planning should also account for broader clinical complexity [42].

Psoriasis is increasingly recognized as a systemic inflammatory condition associated with elevated risks of cardiovascular disease, metabolic syndrome, obesity, and type 2 diabetes, largely driven by chronic low-grade inflammation mediated through the IL-17/23 axis [43]. Recent studies underscore the importance of addressing these cardiometabolic comorbidities in psoriatic patients, as biologic therapies not only improve skin symptoms but also modulate systemic inflammation. A 2024 retrospective study by Ibba et al. demonstrated that risankizumab, an IL-23 inhibitor, maintained long-term effectiveness and safety in patients with moderate-to-severe psoriasis, irrespective of cardiometabolic comorbidities [44]. Similarly, Boskovic et al. reviewed evidence showing that IL-17 inhibitors could reduce coronary plaque burden, and ustekinumab has been associated with improvements in favorable imaging outcomes [45]. These findings highlight the potential for biologic therapies to improve both dermatologic and systemic inflammatory profiles in this population.

This is a large study on the prognostic value of diabetes on adverse cardiovascular events in patients with psoriasis and coronary artery disease. Despite this, some limitations were present in this study. Firstly, since this was a single-center study conducted, the enrolled patients may not fully represent the general psoriasis population, which may limit the generalizability of the findings to other settings. Secondly, this was a retrospective observational study, bias could not be avoided. We attempted to minimize selection bias by including all consecutive eligible patients with complete records during the study period. However, information bias may still be present, as the accuracy of the diagnosis and clinical data relies on existing medical records. Thirdly, the temporal relationship between the diagnoses of psoriasis and diabetes could not be reliably established for all patients. Future prospective studies are needed to explore how the timing of diabetes onset relative to psoriasis may influence cardiovascular risk. Finally, all patient data used in this study were anonymous in accordance with ethical standards, which may affect the comprehensiveness of risk factor assessment and limit more detailed stratified analyses.

## Conclusions

Diabetes in patients with psoriasis and coronary artery disease is linked with cardiovascular adverse events. These findings have important implications for the risk assessment for patients with psoriasis and coronary artery disease. Considering the prevalence of diabetes and psoriasis in the world, our findings have public health implications.

## Electronic Supplementary Material

Below is the link to the electronic supplementary material.

Supplementary Figure 1

## Data Availability

The data used to support the findings of this study are available from the corresponding authors upon request.

## References

[1] Nestle FO, Kaplan DH, Barker J. Psoriasis. N Engl J Med. 2009;361(5):496–509. 19641206 10.1056/NEJMra0804595

[2] Damiani G, Bragazzi NL, Karimkhani Aksut C, Wu D, Alicandro G, McGonagle D, Guo C, Dellavalle R, Grada A, Wong P, et al. The global, regional, and National burden of psoriasis: results and insights from the global burden of disease 2019 study. Front Med. 2021;8:743180. 10.3389/fmed.2021.743180 PMC8716585 34977058

[3] Liu S, Yan Z, Liu Q. The Burden of Psoriasis in China and Global Level from 1990 to 2019: A Systematic Analysis from the Global Burden of Disease Study 2019. *Biomed Res Int* 2022, 2022:3461765. 10.1155/2022/3461765 PMC9560841 36246981

[4] Parisi R, Iskandar IYK, Kontopantelis E, Augustin M, Griffiths CEM, Ashcroft DM. National, regional, and worldwide epidemiology of psoriasis: systematic analysis and modelling study. BMJ. 2020;369:m1590. 32467098 10.1136/bmj.m1590 PMC7254147

[5] Gisondi P, Bellinato F, Girolomoni G, Albanesi C. Pathogenesis of chronic plaque psoriasis and its intersection with Cardio-Metabolic comorbidities. Front Pharmacol. 2020;11:117. 32161545 10.3389/fphar.2020.00117 PMC7052356

[6] Takeshita J, Grewal S, Langan SM, Mehta NN, Ogdie A, Van Voorhees AS, Gelfand JM. Psoriasis and comorbid diseases: epidemiology. J Am Acad Dermatol. 2017;76(3):377–90. 28212759 10.1016/j.jaad.2016.07.064 PMC5731650

[7] Armstrong AW, Harskamp CT, Armstrong EJ. Psoriasis and the risk of diabetes mellitus: a systematic review and meta-analysis. JAMA Dermatology. 2013;149(1):84–91. 23407990 10.1001/2013.jamadermatol.406

[8] Mamizadeh M, Tardeh Z, Azami M. The association between psoriasis and diabetes mellitus: A systematic review and meta-analysis. Diabetes Metab Syndr. 2019;13(2):1405–12. 31336500 10.1016/j.dsx.2019.01.009

[9] Miller IM, Ellervik C, Yazdanyar S, Jemec GB. Meta-analysis of psoriasis, cardiovascular disease, and associated risk factors. J Am Acad Dermatol. 2013;69(6):1014–24. 24238156 10.1016/j.jaad.2013.06.053

[10] Abramczyk R, Queller JN, Rachfal AW, Schwartz SS. Diabetes and psoriasis: different sides of the same Prism. Diabetes Metab Syndr Obes. 2020;13:3571–7. 33116708 10.2147/DMSO.S273147 PMC7548229

[11] Cui P, Li D, Shi L, Yan H, Li T, Liu C, Wang W, Zheng H, Ding N, Li X, et al. Cardiovascular comorbidities among patients with psoriasis: a National register-based study in China. Sci Rep. 2024;14(1):19683. 39181937 10.1038/s41598-024-70707-w PMC11344856

[12] Pan J, Chang X, Wang L, Miao G, Jin Q, Guo N, Zhang J, Lv Y, Wang L. Use of biologics in patients with psoriasis - A retrospective analysis based on real-world data. Skin Res Technol. 2024;30(1):e13550. 38174801 10.1111/srt.13550 PMC10765354

[13] Kommoss KS, Enk A, Heikenwälder M, Waisman A, Karbach S, Wild J. Cardiovascular comorbidity in psoriasis - psoriatic inflammation is more than just skin deep. J Der Deutschen Dermatologischen Gesellschaft = J German Soc Dermatology: JDDG. 2023;21(7):718–25. 10.1111/ddg.15071 37186503

[14] Weng J, Ji L, Jia W, Lu J, Zhou Z, Zou D, Zhu D, Chen L, Chen L, Guo L, et al. Standards of care for type 2 diabetes in China. Diabetes Metab Res Rev. 2016;32(5):442–58. 27464265 10.1002/dmrr.2827 PMC5108436

[15] Elenkova A, Matrozova J, Vasilev V, Robeva R, Zacharieva S. Prevalence and progression of carbohydrate disorders in patients with pheochromocytoma/paraganglioma: retrospective single-center study. Ann Endocrinol (Paris). 2020;81(1):3–10. 32067697 10.1016/j.ando.2020.01.001

[16] Unger T, Borghi C, Charchar F, Khan NA, Poulter NR, Prabhakaran D, Ramirez A, Schlaich M, Stergiou GS, Tomaszewski M, et al. 2020 international society of hypertension global hypertension practice guidelines. Hypertension. 2020;75(6):1334–57. 32370572 10.1161/HYPERTENSIONAHA.120.15026

[17] Cutlip DE, Windecker S, Mehran R, Boam A, Cohen DJ, van Es GA, Steg PG, Morel MA, Mauri L, Vranckx P, et al. Clinical end points in coronary stent trials: a case for standardized definitions. Circulation. 2007;115(17):2344–51. 17470709 10.1161/CIRCULATIONAHA.106.685313

[18] Levey AS, Stevens LA, Schmid CH, Zhang YL, Castro AF 3rd, Feldman HI, Kusek JW, Eggers P, Van Lente F, Greene T, et al. A new equation to estimate glomerular filtration rate. Ann Intern Med. 2009;150(9):604–12. 19414839 10.7326/0003-4819-150-9-200905050-00006 PMC2763564

[19] Xu N, Tang XF, Yao Y, Zhao XY, Chen J, Gao Z, Qiao SB, Yang YJ, Gao RL, Xu B, et al. Association of plasma Lipoprotein(a) with Long-Term adverse events in patients with chronic kidney disease who underwent percutaneous coronary intervention. Am J Cardiol. 2018;122(12):2043–8. 30477725 10.1016/j.amjcard.2018.04.058

[20] Gelfand JM, Neimann AL, Shin DB, Wang X, Margolis DJ, Troxel AB. Risk of myocardial infarction in patients with psoriasis. JAMA. 2006;296(14):1735–41. 17032986 10.1001/jama.296.14.1735

[21] Mehta NN, Azfar RS, Shin DB, Neimann AL, Troxel AB, Gelfand JM. Patients with severe psoriasis are at increased risk of cardiovascular mortality: cohort study using the general practice research database. Eur Heart J. 2010;31(8):1000–6. 20037179 10.1093/eurheartj/ehp567 PMC2894736

[22] Kwon OC, Han K, Chun J, Kim R, Hong SW, Kim JH, Youn YH, Park H, Park MC. Effects of immune-mediated inflammatory diseases on cardiovascular diseases in patients with type 2 diabetes: a nationwide population-based study. Sci Rep. 2022;12(1):11548. 35798796 10.1038/s41598-022-15436-8 PMC9262934

[23] Piaserico S, Orlando G, Messina F. Psoriasis and cardiometabolic diseases: shared genetic and molecular pathways. Int J Mol Sci 2022, 23(16). 10.3390/ijms23169063 PMC9409274 36012327

[24] Wang H, Wang Z, Rani PL, Fu X, Yu W, Bao F, Yu G, Li J, Li L, Sun L, et al. Identification of PTPN22, ST6GAL1 and JAZF1 as psoriasis risk genes demonstrates shared pathogenesis between psoriasis and diabetes. Exp Dermatol. 2017;26(11):1112–7. 28603863 10.1111/exd.13393

[25] Wolf N, Quaranta M, Prescott NJ, Allen M, Smith R, Burden AD, Worthington J, Griffiths CE, Mathew CG, Barker JN, et al. Psoriasis is associated with pleiotropic susceptibility loci identified in type II diabetes and Crohn disease. J Med Genet. 2008;45(2):114–6. 17993580 10.1136/jmg.2007.053595

[26] Brazzelli V, Maffioli P, Bolcato V, Ciolfi C, D’Angelo A, Tinelli C, Derosa G. Psoriasis and diabetes, a dangerous association: evaluation of insulin resistance, lipid abnormalities, and cardiovascular risk biomarkers. Front Med. 2021;8:605691. 10.3389/fmed.2021.605691 PMC8021695 33834030

[27] Ramos S, Daya S, Crowther NJ, Pillay L, Tikly M, Goolam Mahyoodeen N. Prevalence and predictors of abdominal aorta calcification in patients with Psoriasis-A case control study. Front Med. 2022;9:890195. 10.3389/fmed.2022.890195 PMC9280304 35847770

[28] Criqui MH, Denenberg JO, McClelland RL, Allison MA, Ix JH, Guerci A, Cohoon KP, Srikanthan P, Watson KE, Wong ND. Abdominal aortic calcium, coronary artery calcium, and cardiovascular morbidity and mortality in the Multi-Ethnic study of atherosclerosis. Arterioscler Thromb Vasc Biol. 2014;34(7):1574–9. 24812323 10.1161/ATVBAHA.114.303268 PMC4153597

[29] Wilson PW, Kauppila LI, O’Donnell CJ, Kiel DP, Hannan M, Polak JM, Cupples LA. Abdominal aortic calcific deposits are an important predictor of vascular morbidity and mortality. Circulation. 2001;103(11):1529–34. 11257080 10.1161/01.cir.103.11.1529

[30] Committee ADAPP. 10. Cardiovascular disease and risk management: standards of medical care in Diabetes-2022. Diabetes Care. 2022;45(Suppl 1):S144–74. 34964815 10.2337/dc22-S010

[31] Lu J, Chen K, Chen W, Liu C, Jiang X, Ma Z, Li D, Shen Y, Tian H. Association of Serum Homocysteine with Cardiovascular and All-Cause Mortality in Adults with Diabetes: A Prospective Cohort Study. *Oxid Med Cell Longev* 2022, 2022:2156483. 10.1155/2022/2156483 PMC9578792 36267812

[32] Rehman T, Shabbir MA, Inam-Ur-Raheem M, Manzoor MF, Ahmad N, Liu ZW, Ahmad MH, Siddeeg A, Abid M, Aadil RM. Cysteine and homocysteine as biomarker of various diseases. Food Sci Nutr. 2020;8(9):4696–707. 32994931 10.1002/fsn3.1818 PMC7500767

[33] Djuric D, Jakovljevic V, Zivkovic V, Srejovic I. Homocysteine and homocysteine-related compounds: an overview of the roles in the pathology of the cardiovascular and nervous systems. Can J Physiol Pharmacol. 2018;96(10):991–1003. 30130426 10.1139/cjpp-2018-0112

[34] Steinberg D. The LDL modification hypothesis of atherogenesis: an update. J Lipid Res. 2009;50(SupplSuppl):S376–381. 19011257 10.1194/jlr.R800087-JLR200 PMC2674707

[35] Jung E, Kong SY, Ro YS, Ryu HH, Shin SD. Serum cholesterol levels and risk of cardiovascular death: A systematic review and a Dose-Response Meta-Analysis of prospective cohort studies. Int J Environ Res Public Health 2022, 19(14). 10.3390/ijerph19148272 PMC9316578 35886124

[36] Zheng Q, Kuai L, Jiang W, Qiang Y, Wei L, Chen S, Li B, Wang R. Clinical feature, lifestyle behavior and Non-Communicable diseases comorbidities among psoriasis patients in shanghai: gender disparity analysis based on a Cross-Sectional study. Clin Cosmet Invest Dermatology. 2022;15:2751–62. 10.2147/CCID.S393697 PMC9762258 36545501

[37] Ungprasert P, Raksasuk S. Psoriasis and risk of incident chronic kidney disease and end-stage renal disease: a systematic review and meta-analysis. Int Urol Nephrol. 2018;50(7):1277–83. 29644523 10.1007/s11255-018-1868-z

[38] Buja A, Miatton A, Cozzolino C, Brazzale AR, Lo Bue R, Mercuri SR, Proft FN, Kridin K, Cohen AD, Damiani G. The prevalent comorbidome at the onset of psoriasis diagnosis. Dermatology Therapy. 2023;13(9):2093–105. 37542678 10.1007/s13555-023-00986-0 PMC10442308

[39] Damiani G, Amerio P, Bardazzi F, Carrera CG, Conti A, Cusano F, Dapavo P, DeSimone C, El Hachem M, Fabbrocini G, et al. Real-World experience of methotrexate in the treatment of skin diseases: an Italian Delphi consensus. Dermatology Therapy. 2023;13(6):1219–41. 37210684 10.1007/s13555-023-00930-2 PMC10200012

[40] Damiani G, Pacifico A, Scoditti E, di Gregorio S, Del Fabbro M, Cozzolino C, Buja A, Mercuri SR, Bianchi VG, Grada A, et al. Circadian oscillations of minimal erythema dose (MED) are also influenced by diet in patients with psoriasis: A chronomedical study. Dermatology Therapy. 2023;13(10):2229–46. 37573289 10.1007/s13555-023-00987-z PMC10539244

[41] Pacifico A, Conic RRZ, Cristaudo A, Garbarino S, Ardigò M, Morrone A, Iacovelli P, di Gregorio S, Pigatto PDM, Grada A et al. Diet-Related Phototoxic Reactions in Psoriatic Patients Undergoing Phototherapy: Results from a Multicenter Prospective Study. *Nutrients* 2021, 13(9). 10.3390/nu13092934 PMC8470626 34578812

[42] Damiani G, Pacifico A, Ricciardi S, Corazza V, Trigos D, Fiore M, Guarneri C. Management of systemic Anti-psoriatic drugs in psoriasis patients with concurrent paraplegia or tetraplegia: insights from a 6-Year multicenter, retrospective observational study. Dermatology Therapy. 2025;15(2):427–36. 39849247 10.1007/s13555-025-01338-w PMC11832867

[43] Trovato E, Rubegni P, Prignano F. Place in therapy of anti-IL-17 and 23 in psoriasis according to the severity of comorbidities: a focus on cardiovascular disease and metabolic syndrome. Expert Opin Biol Ther. 2022;22(12):1443–8. 35726639 10.1080/14712598.2022.2093106

[44] Ibba L, Di Giulio S, Gargiulo L, Facheris P, Perugini C, Costanzo A, Narcisi A, Valenti M. Long-term effectiveness and safety of Risankizumab in patients with moderate-to-severe psoriasis with and without cardiometabolic comorbidities: a single-center retrospective study. J Dermatolog Treat. 2024;35(1):2425029. 39510528 10.1080/09546634.2024.2425029

[45] Boskovic S, Borriello S, D’Ascenzo F, Sciamarrelli N, Rosset F, Mastorino L, Paolo D, Bocchino PP, De Filippo O, Ribero S, et al. Effectiveness of biological therapy in reducing psoriasis-related cardiovascular risk. Expert Opin Biol Ther. 2024;24(4):217–9. 38557408 10.1080/14712598.2024.2337242

