# Supplementary figures and images for: Diabetes is associated with a poor prognosis in patients with psoriasis and coronary artery disease
Source: BMC Endocr Disord. 2025 Jul 14;25:174. doi: 10.1186/s12902-025-01996-z (PMC12257814; doi:10.1186/s12902-025-01996-z)

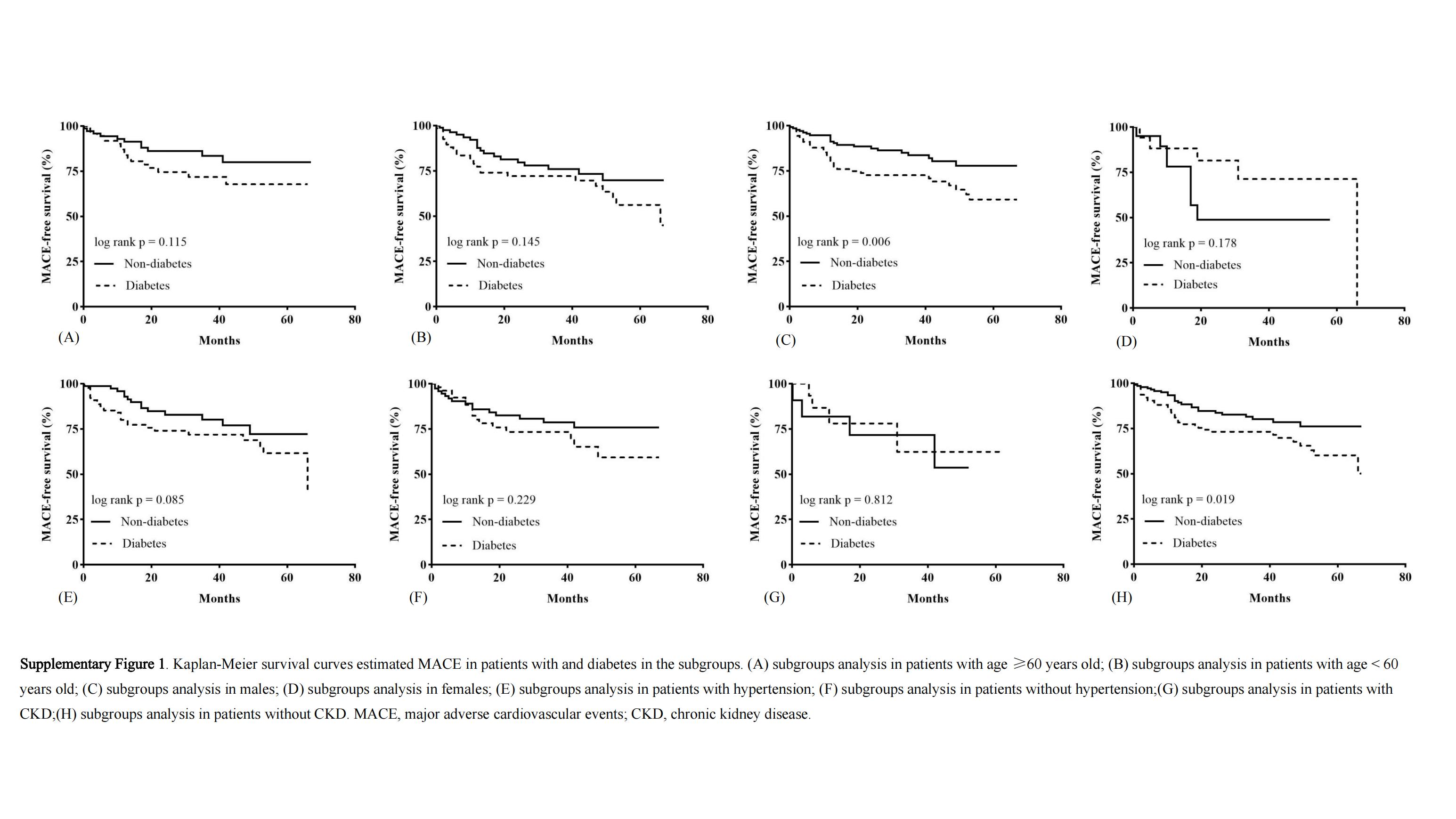

Supplement: Supplementary file 1 — Supplementary Figure 1 [file 12902_2025_1996_MOESM1_ESM.jpg]
